# Supplementary material for: HMGB1 B‑Box Domain Associates Promote Protein–Polyelectrolyte Interactions
Source: J Phys Chem B. 2025 Sep 6;129(37):9293–303. doi: 10.1021/acs.jpcb.5c02892 (PMC12451656; doi:10.1021/acs.jpcb.5c02892)
Supplement: Supplementary file 1 [file jp5c02892_si_001.pdf]

SUPPLEMENTARY MATERIAL

HMGB1 B-box Domain Associates Promote Protein-Polyelectrolyte Interactions.

Marten Kagelmacher[a,b], Marina Pigaleva[*,a], Ricardo Zarate[a], Leïla Bechtella[a, c], Kevin Pagel[a, c], Beate Koksch[a], Jens Dernedde[b], Andreas Herrmann[a], Thomas Risse[a].

[a] Institute of Chemistry and Biochemistry, Freie Universität Berlin, 14195 Berlin, Germany

[b] Institut für Laboratoriumsmedizin, Klinische Chemie und Pathobiochemie, Charité—Universitätsmedizin Berlin, 13353 Berlin, Germany

[c] Fritz Haber Institute of the Max Planck Society, 14195 Berlin, Germany

[*]Corresponding author: marina.pigaleva@fu-berlin.de

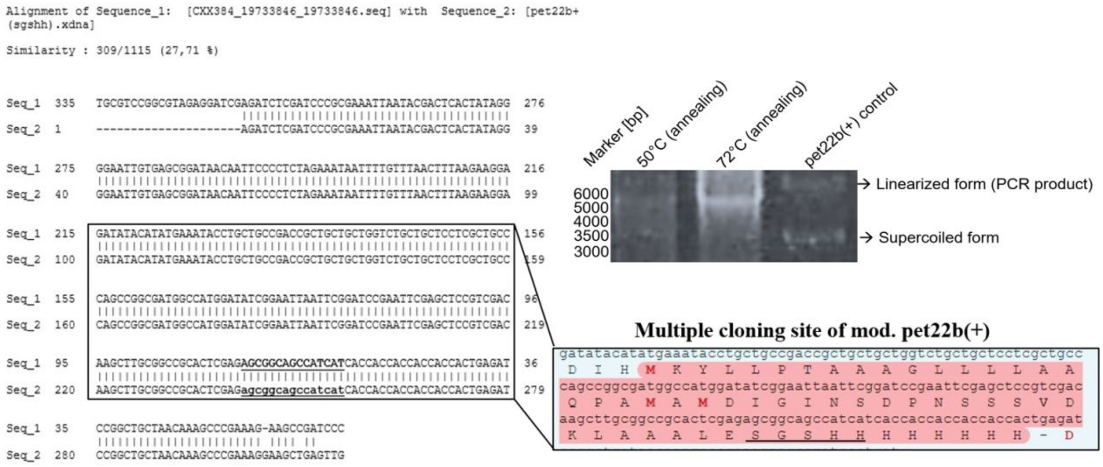

Figure S1. Cloning results of the C-terminal modification of the pet22 b(+) vector. The C-terminal insertion of a Serine-Glycine-Serine (SGS) and two histidine´s was achieved by PCR and blunt end cloning. Successful cloning was verified by sequencing and the inserted amino acids are underlined.

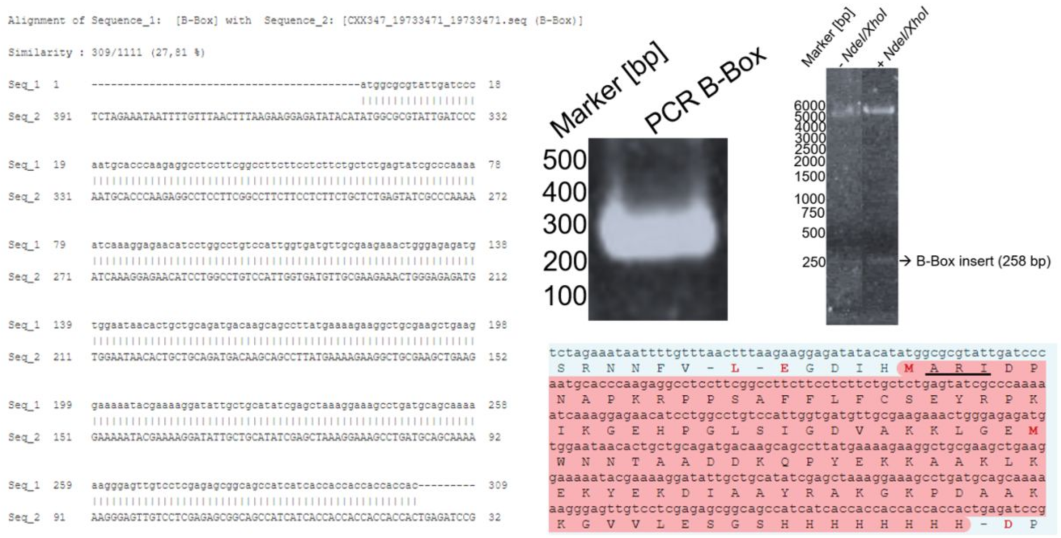

Figure S2. Cloning of human B-box into mod. pet22 b(+) vector. The sequence of the human B-box protein from HMGB1 was cloned into the mod. pet22 b(+) vector by ELIC. Successful cloning was verified by sequencing and the corresponding amino acid sequence was highlighted in pink. The three artificial amino acids Alanine-Arginin-Isoleucin are underlined.

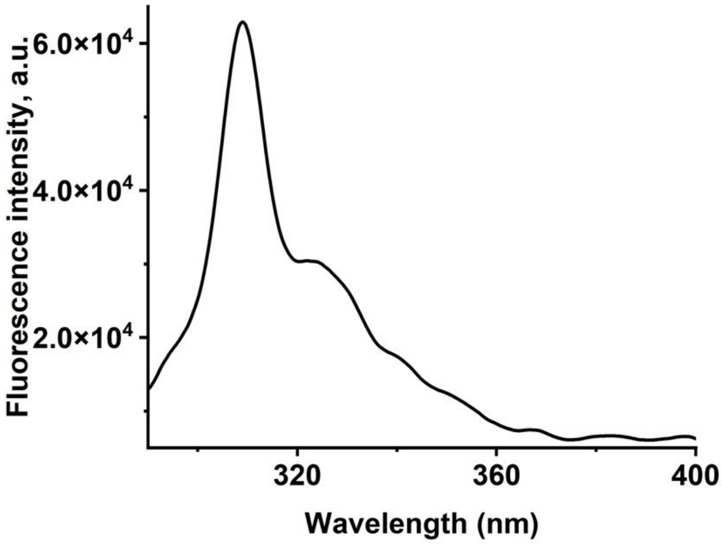

Figure S3. Baseline (buffer solution, 25 mM Tris, 150 mM NaCl, pH 6.9) for the fluorescence experiments. The Raman scattering peak is visible at around 310 nm.

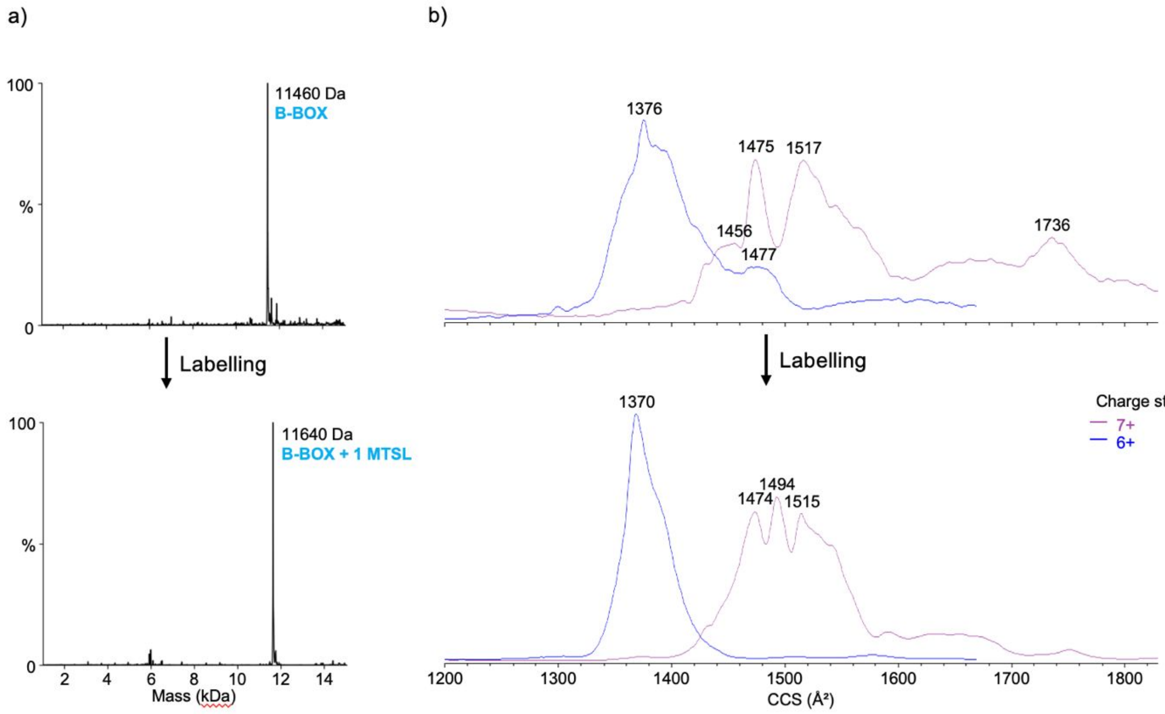

Figure S4. a) Native ion mobility-mass spectrometry (IM-MS) experiments on the unfiltered B-box domain before and after MTSL labeling. a) Deconvoluted mass spectra and b) Extracted ion mobilograms of B-BOX charge states 6+ and 7+, at m/z 1941.6 and m/z 1664.4, respectively.

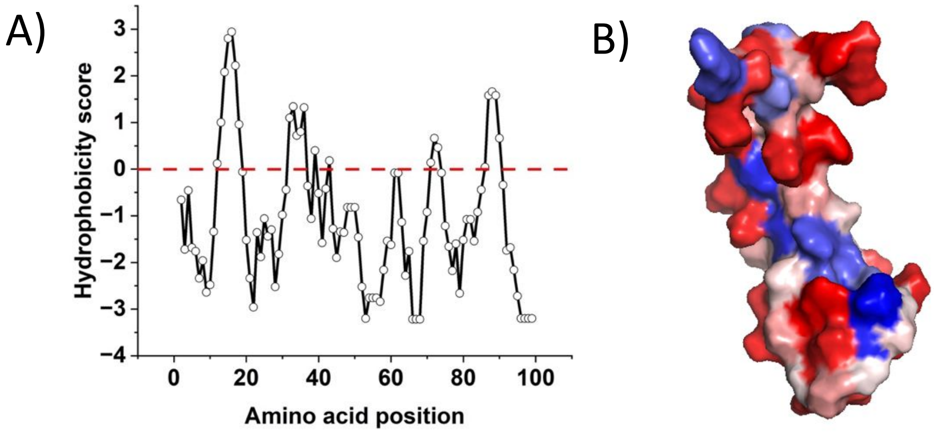

Figure S5. A) Hydrophobicity of the B-Box domain along the chain. Kyte-Dolittle scale, with window size 7, relative weight for window edges - 100 % and weight variation model – linear.[1] B) 3D presentation of hydrophilic (blue) and hydrophobic (red) regions on the protein surface.

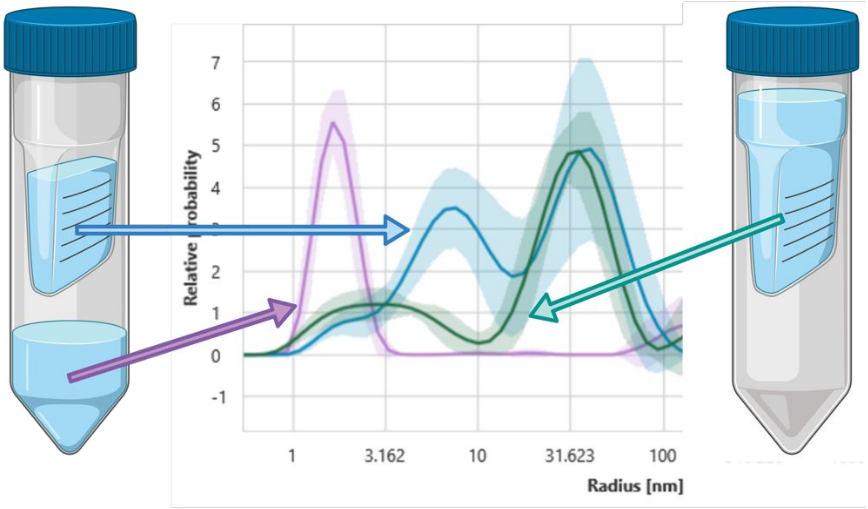

Figure S6. Scheme of the filtration procedure to obtain a monomeric B-Box protein solution, and the corresponding DLS spectra of the unfiltered protein solution (green line), solution from the bottom of the filter (species < 30 kDa; violet line), solution from the top of the filter (species > 30 kDa; blue line).

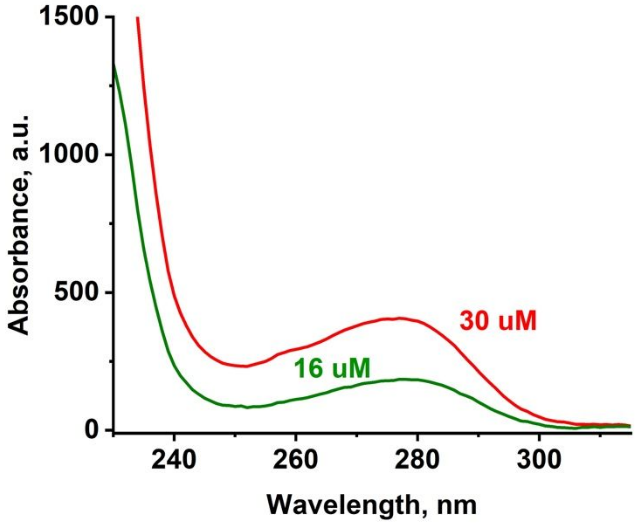

Figure S7. UV-vis spectra of the B-Box solution before (red line) and after (dark green line) the filtration through 30 kDa membrane filter and corresponding concentration measured according to the intensity at 280 nm.

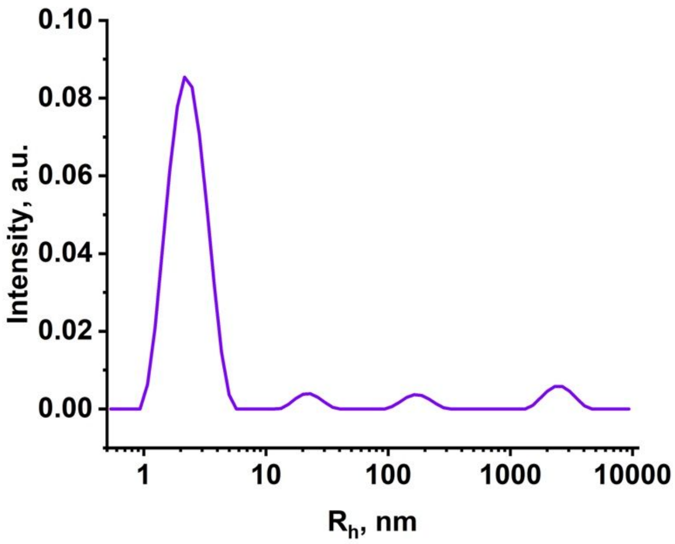

Figure S8. Hydrodynamic radius (R_h) of A-box HMGB1 protein domain in buffer solution (25 mM Tris-HCl pH 6.9, 150 mM NaCl).

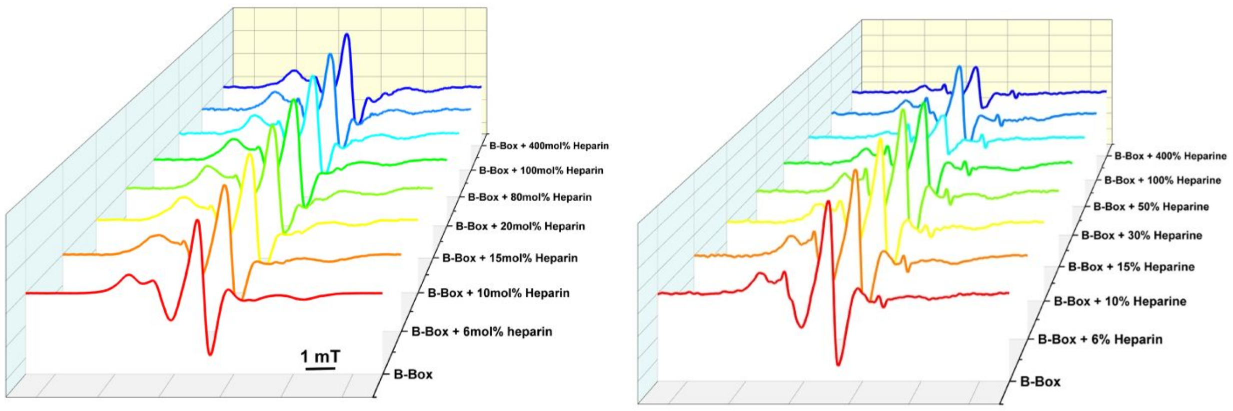

Figure S9. EPR spectra of HMGB1 B-box domain protein in the self-associated (left) and monomeric (right) states after gradual addition of heparin.

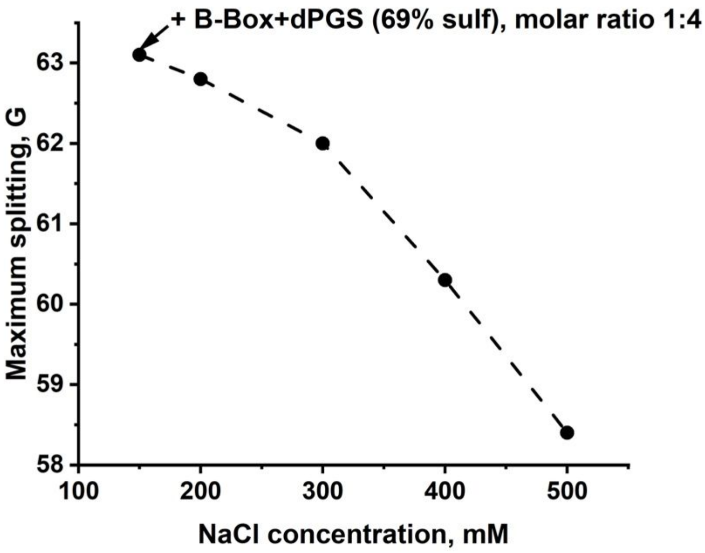

Figure S10. $2A_{zz}$ value obtained from the EPR spectra of B-Box protein interacting with polyglycerolsulfate at different ionic strength of the solution. The complete disruption of interactions is visible at 500 mM salt concentration.

---

[1] Kyte, J. and Doolittle, R.F., 1982. A simple method for displaying the hydropathic character of a protein. *Journal of molecular biology*, *157*(1), pp.105-132.
